# Can diffusion-weighted imaging add information in the evaluation of breast lesions considered suspicious on magnetic resonance imaging?

**DOI:** 10.1590/0100-3984.2016.0089

**Published:** 2017

**Authors:** Camila Souza Guatelli, Almir Galvão Vieira Bitencourt, Cynthia Aparecida Bueno de Toledo Osório, Luciana Graziano, Alessandra Araújo de Castro, Juliana Alves de Souza, Elvira Ferreira Marques, Rubens Chojniak

**Affiliations:** 1 MSc, MD, Radiologist at the A.C.Camargo Cancer Center, São Paulo, SP, Brazil.; 2 PhD, MD, Radiologist at the A.C.Camargo Cancer Center, São Paulo, SP, Brazil.; 3 PhD, MD, Pathologist at the A.C.Camargo Cancer Center, São Paulo, SP, Brazil.; 4 MD, Resident in the Imaging Department of the A.C.Camargo Cancer Center, São Paulo, SP, Brazil.; 5 MD, Radiologist at the A.C.Camargo Cancer Center, São Paulo, SP, Brazil.; 6 MD, Radiologist, Head of the Department of Breast Imaging, A.C.Camargo Cancer Center, São Paulo, SP, Brazil.; 7 PhD, MD, Radiologist, Director of the Imaging Department of the A.C.Camargo Cancer Center, São Paulo, SP, Brazil.

**Keywords:** Breast neoplasms, Mammography, Ultrasonography, mammary, Magnetic resonance imaging, Neoplasias da mama, Mamografia, Ultrassonografia mamária, Ressonância magnética

## Abstract

**Objective:**

To assess the role of diffusion-weighted imaging (DWI) in the evaluation of breast lesions classified as suspicious on magnetic resonance imaging (MRI), correlating the findings with the results of the histological analysis.

**Materials and Methods:**

This was a retrospective, descriptive study based on a review of the medical records of 215 patients who were submitted to MRI with DWI before undergoing biopsy at a cancer center. Apparent diffusion coefficient (ADC) values were calculated for each lesion, and the result of the histological analysis was considered the gold standard.

**Results:**

The mean age was 49 years. We identified 252 lesions, 161 (63.9%) of which were found to be malignant in the histological analysis. The mean ADC value was higher for the benign lesions than for the malignant lesions (1.50 × 10^–3^ mm^2^/s vs. 0.97 × 10^−3^ mm^2^/s), the difference being statistically significant (*p* < 0.001). The ADC cut-off point with the greatest sensitivity and specificity on the receiver operating characteristic curve was 1.03 × 10^−3^ mm^2^/s. When the DWI and conventional MRI findings were combined, the accuracy reached 95.9%, with a sensitivity of 95.7% and a specificity of 96.4%.

**Conclusion:**

The use of DWI could facilitate the characterization of breast lesions, especially those classified as BI-RADS 4, increasing the specificity and diagnostic accuracy of MRI.

## Abstract (pt)

**Objetivo:**

Avaliar o papel da sequência em difusão na avaliação de lesões mamárias suspeitas na ressonância magnética (RM), correlacionando seus achados com os resultados histológicos.

**Materiais e Métodos:**

Foi realizado estudo retrospectivo, descritivo, baseado na análise de prontuários médicos de 215 pacientes que realizaram RM com sequência em difusão e que foram submetidas a biópsia em um centro de referência oncológico. Foi calculado o valor do coeficiente de difusão aparente (ADC – *apparent diffusion coefficient*) para cada lesão e o resultado histológico foi considerado como padrão ouro.

**Resultados:**

A idade média das pacientes foi 49 anos. Foram identificadas 252 lesões, e destas, 161 (63,9%) eram lesões malignas na avaliação histológica. A média obtida do valor do ADC nas lesões benignas (1,50 × 10^–3^ mm^2^/s) foi superior à média das lesões malignas (0,97 × 10^–3^ mm^2^/s), com significância estatística (*p* < 0,001). O ponto de corte com maior sensibilidade e especificidade pela curva *receiver operating characteristic* foi 1,03 × 10^–3^ mm^2^/s. Com a combinação da difusão com os achados da RM, a acurácia chegou a 95,9%, com sensibilidade de 95,7% e especificidade de 96,4%.

**Conclusão:**

O uso da sequência em difusão pode auxiliar na caracterização das lesões mamárias, principalmente daquelas classificadas como BI-RADS 4, aumentando a especificidade e a acurácia diagnóstica da RM.

## INTRODUCTION

Because it provides information regarding the vascularization of the breast parenchyma, magnetic resonance imaging (MRI) has greater sensitivity in the detection of breast cancer than do mammography and ultrasound, making it an important tool in the screening of high-risk patients with dense breasts^(1)^. MRI also shows greater accuracy in assessing the extent of the disease and in detecting additional lesions in the contralateral breast during staging, thereby improving surgical and treatment planning^(2-4)^. New MRI techniques have been developed with the objective of adding functional information to the morphological and kinetic analysis, in order to improve the specificity of the method. Among such techniques, diffusion-weighted imaging (DWI) is the one that is currently being most widely studied^(5)^.

DWI sequences use gradients that are sensitive to the movement of water molecules^(6)^. Thus, DWI demonstrates differences in the movement of water molecules in tissues. In malignant tumors, increased cell proliferation results in greater cell density, creating more barriers for the diffusion of water molecules (restricted diffusion), which manifests as hypointense signals on DWI. In contrast, benign tumors show lower cell density and a larger extracellular space, thus presenting fewer obstacles to the diffusion of water molecules. Images obtained from apparent diffusion coefficient (ADC) mapping can be analyzed qualitatively and quantitatively. Given these characteristics, DWI appears to be a useful tool for differentiating between benign and malignant lesions, increasing the specificity of MRI, providing important information for treatment planning and follow-up, as well as allowing the response to neoadjuvant chemotherapy to be evaluated^(7-10)^.

The objective of this study was to evaluate the role of DWI in the evaluation of breast lesions classified as suspicious on conventional MRI. We also attempted to determine whether DWI findings correlate with those obtained in histological and immunohistochemical analyses.

## MATERIALS AND METHODS

This was a retrospective descriptive study based on the analysis of medical records and data collected from a group of patients who underwent MRI with DWI between August 2010 and December 2013 at a referral center for cancer. The study was approved by the research ethics committee of the institution.

We initially selected 238 patients in whom MRI scans identified lesions, who also underwent MRI with DWI, and who were subsequently submitted to percutaneous or surgical biopsy. Of those, 23 were excluded because the DWI evaluation was compromised by movement artifacts or other technical issues. Therefore, the final study sample consisted of 215 patients, in whom a total of 252 lesions were identified.

All MRI scans were performed in a 1.5 T scanner (Signa HDxt; GE Healthcare, Waukesha, WI, USA) with a dedicated breast coil. DWI was performed before the dynamic phase of contrast enhancement, with array spatial sensitivity encoding technique echo-planar imaging in the axial plane (TR/TE, 4000/94; matrix, 192 × 192; signal average, 3; slice thickness, 3 mm; distance factor, 20%). The diffusion-sensitizing gradients were applied in two orthogonal directions, with two b values: 0 and 750 s/mm^2^.

The MRI scans of the breasts were reviewed on a dedicated workstation (Advantage Workstation 4; GE Healthcare) by a radiologist with experience in breast imaging. The lesions were evaluated and classified according to the criteria of the Breast Imaging Reporting and Data System (BI-RADS) for MRI, 5th edition, defining the morphological aspects by the type of enhancement (nodular or non-nodular); its distribution, shape, and contours; and the pattern of intravenous contrast uptake in the subsequent dynamic evaluation.

The DWI sequences were post-processed with commercial software (FuncTool 7.4.01d; GE Healthcare). Qualitative and quantitative evaluations were based on the ADC. For the qualitative evaluation, we used gray-scale ADC maps, classifying lesions with restricted diffusion as those in which there was high signal intensity on DWI and signal loss on the ADC map. For the quantitative evaluation, we calculated the mean ADC, selecting the region of interest (ROI) within the lesion, avoiding areas of necrosis and cystic degeneration. The ADC values were calculated by using the following formula:

$$\mathrm{ADC}=-\left(1/b\right)\ln\left(S2/S1\right)$$

where *S2* and *S1* are the intensities of the 0 and 750 s/mm^2^*b* values, respectively^(5)^.

Twenty lesions were excluded from the quantitative analysis because it was not possible to calculate the value of the ADC due to limitations of image recovery on the workstation. Therefore, only the qualitative evaluation of the diffusion was performed in those cases.

Histological data were collected through analysis of the surgical specimen, when available, or of percutaneous biopsy material. Histological types were reported according to the tumor classification system of the World Health Organization^(11)^ and the Nottingham (Elston-Ellis) modification^(12)^ of the Scarff-Bloom-Richardson grading system.

In the statistical analysis, the normality of the variables was tested by the Shapiro-Wilk test and the associations were tested by Pearson’s chi-square test or Fisher’s exact test, as appropriate. Continuous variables were evaluated using unpaired Student’s *t*-test and analysis of variance, together with non-parametric Mann-Whitney and Kruskal-Wallis tests, all of them with a significance level of 5%, values of *p* < 0.05 therefore being considered statistically significant. To evaluate the diagnostic validity of DWI, the histological result was considered the reference. To determine the cut-off ADC values that best classify the lesions as suspected malignancy, we used receiver operating characteristic (ROC) curves. To analyze the signal increase on DWI as a predictive factor of tumor malignancy, we used a logistic regression model including the ADC and diffusion restriction variables. The data collected were compiled in a database created in the program Excel for Windows, and the statistical analysis was carried out with the software Stata, version 11 SE (StataCorp LLC, College Station, TX, USA), the Statistical Package for the Social Sciences, version 16.0 (SPSS Inc., Chicago, IL, USA), and MedCalc, version 15.6.1 (MedCalc Software, Ostend, Belgium).

## RESULTS

Of the 215 patients included in the study, only one was male. The mean ± standard deviation for age was 49 ± 12 years (range, 23–88 years). The majority (75.8%) of the patients evaluated were ≥ 40 years of age, 61 (28.4%) had a positive family history of breast cancer, and 19 (8.8%) had a personal history of breast cancer.

Among the 215 patients included, there were a total of 252 lesions, with a mean size of 27 ± 22 mm (range, 4–117 mm). Of the 252 lesions, 210 (83.3%) showed nodular enhancement, 40 (15.8%) showed non-nodular enhancement, and 2 (0.8%) showed no enhancement.

In 106 (42.1%) of the 252 lesions evaluated, percutaneous biopsy alone was performed, whereas surgical biopsy alone was performed in 71 (28.2%) and both were performed in 75 (29.8%). The biopsy results indicated that 91 (36.1%) of the lesions were benign and 161 (63.9%) were malignant. Benign lesions included but were not limited to fibroadenoma (*n* = 34), papilloma (*n* = 12), stromal fibrosis (*n* = 11), fibrocystic change (*n* = 8), and atypical lobular hyperplasia (*n* = 1), the last being the only lesion that showed atypia. Among the malignant lesions, ductal carcinoma in situ (DCIS) accounted for 11 and invasive carcinoma accounted for 150, of which 121 were classified as invasive carcinoma of no special type (IC-NST).

Table 1 shows the BI-RADS categories, MRI morphological features, and MRI dynamic aspects, in relation to the histological results. All of the lesions classified as BI-RADS 2 or 3 were found to be benign in the histopathological analysis. Of the BI-RADS 4 lesions, 74.5% were found to be benign. Among the 30 lesions classified as BI-RADS 5, only one was found to be benign, the histological result in that case being complex sclerosing lesion. We found that, for the distinction between benign and malignant lesions, the BI-RADS MRI classification had a sensitivity of 100%, a specificity of 54.9%, a positive predictive value (PPV) of 79.7%, a negative predictive value (NPV) of 100%, and an accuracy of 83.7%.

**Table 1:** Relationship between the morphological/dynamic characteristics of the lesions and the histopathological findings.

	Benign			Malignant		
	(*n* = 91)			( *n* = 161)		
Characteristics of the lesions	N	(%)		N	(%)	*P*
BI-RADS						
2	2	(100)*		0	(0.0)	NP
3	48	(100)		0	(0.0)	
4	40	(74.1)		14	(25.9)	
5	1	(2.9)		33	(97.1)	
6	0	(0.0)		114	(99.1)	
Nodular lesions	74	(35.2)		136	(64.8)	
Form						
Irregular	6	(6.9)		81	(93.1)	< 0.001
Regular	68	(55.3)		55	(44.7)	
Borders						
Spiculated	1	(3.1)		31	(96.9)	< 0.001
Irregulars	16	(14.7)		93	(85.3)	
Regulars	58	(85.3)		10	(14.7)	
Internal enhancement						
Heterogeneous	24	(17.8)		111	(82.2)	< 0.001
Homogeneous	44	(78.6)		12	(21.4)	
Peripheral	6	(26.1)		17	(73.9)	
Contrast uptake phase						
Persistently enhancing	59	(64.1)		33	(35.9)	< 0.001
Plateau	11	(30.6)		25	(69.4)	
Washout	6	(6.5)		87	(93.5)	
Non-nodular enhancement	16	(40.0)		24	(60.0)	
Distribution						
Focal	9	(100)		0	(0.0)	NP
Linear or ductal	2 0	(100)		0	(0.0)	
Regional		(0.0)		6	(100)	
Segmental	5	(21.7)		18	(78.3)	

* Lesions that did not show enhancement on MRI and were therefore not evaluated for their morphological and dynamic characteristics. NP, not possible (to test the association).

**Figure 1:**
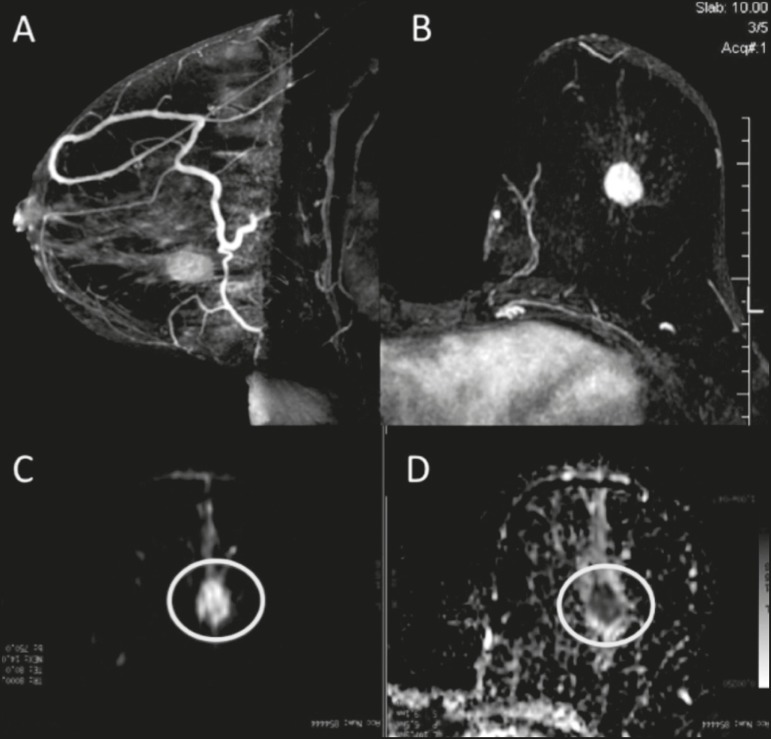
A 64-year-old woman with a nodule in the left breast. Maximum intensity projection reconstructions of the post-contrast subtraction sequence in the sagittal and axial planes (A and B, respectively), showing a circumscribed nodule with heterogeneous enhancement, which presented high signal intensity in the DWI sequence (C) and low signal intensity on the ADC map (D), with an ADC value of 0.74 × 10^−3^ mm^2^/s. The histological findings were consistent with a diagnosis of invasive carcinoma of non-special type.

**Figure 2:**
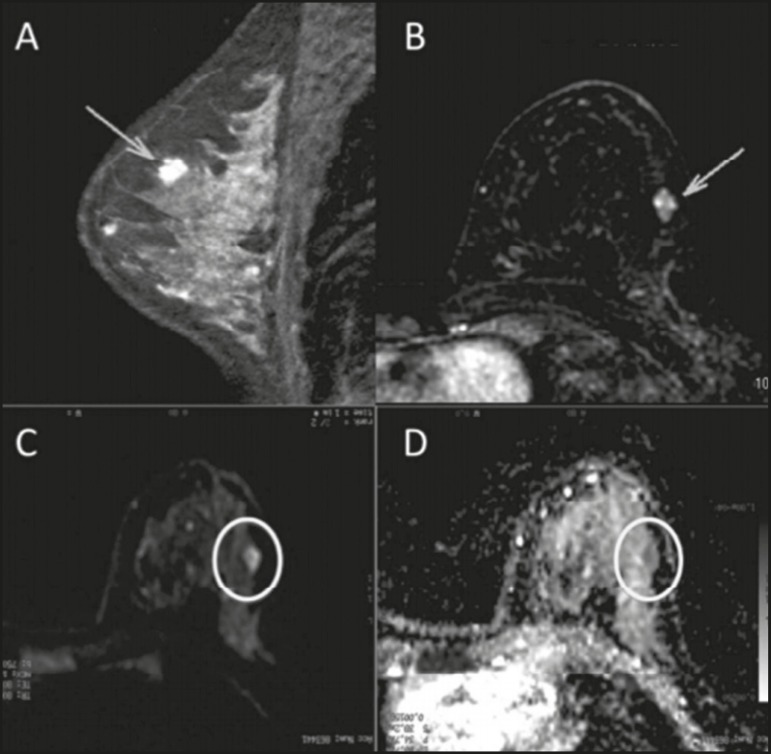
A 35-year-old female with invasive carcinoma in the right breast and presenting with a nodule in the left breast on MRI. Contrast-enhanced T1-weighted images, in the sagittal plane (A) and with subtraction in the axial plane (B), showing a circumscribed nodule with heterogeneous enhancement, high signal intensity in the DWI sequence (C), low signal intensity on the ADC map (D), and an ADC value of 1.32 × 10^−3^ mm^2^/s. The histological findings were consistent with a diagnosis of fibroadenoma.

In the qualitative analysis, DWI showed restricted diffusion in 216 (85.7%) of the 252 lesions. In the quantitative analysis, the mean ADC value was 1.13 ± 0.38 × 10^−3^ mm^2^/s (range 0.38–2.69 10^−3^ mm^2^/s). Figures 1 and 2 depict examples of the lesions evaluated.

The comparison between the qualitative DWI analysis and the histopathological findings showed that the majority (72.7%) of the lesions that presented restricted diffusion were found to be malignant (*p* < 0.001). For the distinction between benign and malignant lesions, the DWI qualitative analysis showed a sensitivity of 97.5%, a specificity of 35.2%, a PPV of 72.7%, an NPV of 88.9%, and an accuracy of 75.0%.

The quantitative analysis of diffusion was obtained by calculating the ADC value for each lesion and then comparing it with the histopathological result. The mean ADC value was higher for the benign lesions than for the malignant lesions (1.50 ± 0.35 × 10^−3^ mm^2^/s vs. 0.97 ± 0.27 × 10^−3^ mm^2^/s), the difference being statistically significant (*p* < 0.001). The histological results were further consolidated into three groups: invasive carcinomas (*n* = 149), with a mean ADC of 0.95 × 10^−3^ mm^2^/s; precursor lesions (*n* = 12), with a mean ADC of 1.24 × 10^−3^ mm^2^/s, among which the histopathological diagnosis was DCIS in 11 and atypical lobular hyperplasia in one; and benign lesions (*n* = 71), with a mean ADC of 1.49 × 10^−3^ mm^2^/s. The difference among the means was significant (*p* < 0.001).

Among the invasive carcinomas, the mean ADC was not found to be associated with the histological or immunohistochemical findings related to tumor aggressiveness (Table 2). Among the lesions classified as DCIS, there was also no statistical difference in terms of the nuclear grade.

**Table 2:** Relationship between the mean ADC values and the histological/immunohistochemical grade in invasive carcinomas.

Histological and immunohistochemical findings in invasive carcinomas	Mean ADC	*P*
Histological grade		
1	1.03	0.14
2	0.90	
3	0.96	
Nuclear grade		
1	0.81	0.83
2	0.96	
3	0.93	
Immunophenotype		
Her-2	0.94	0.97
Luminal A	0.95	
Luminal B	0.93	
Triple negative or basal	0.94	
Estrogen receptor		
Negative	1.00	0.15
Positive	0.92	
Progesterone receptor		
Negative	0.88	0.15
Positive	0.96	
HER2		
Negative	0.93	0.52
Positive	0.95	
Ki-67 index		
1-20%	0.94	0.43
21-30%	0.88	
> 30%	0.96	

**Figure 3:**
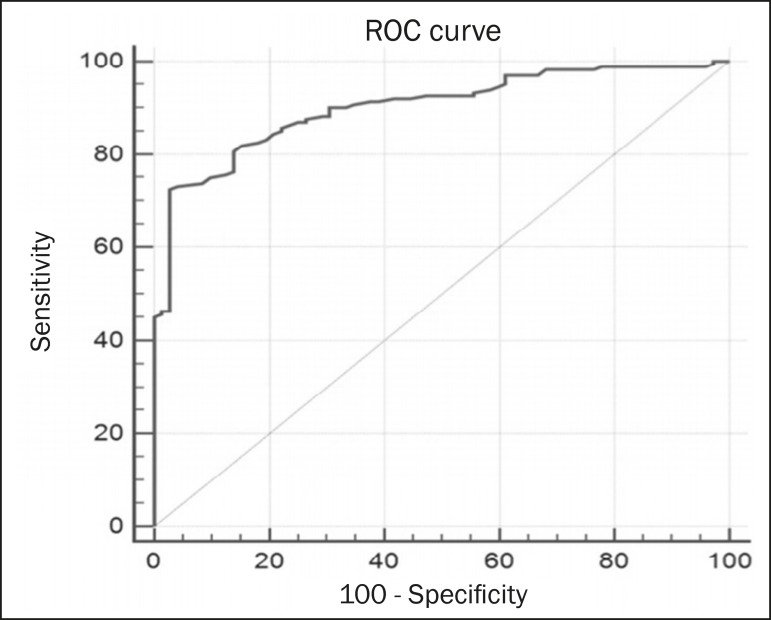
ROC curve to evaluate the diagnostic accuracy of the ADC value in the diagnosis of breast lesions.

Analysis of the ROC curve (Figure 3) showed an area under the curve of 0.901 (standard error: 0.0199; 95% confidence interval: 0.855–0.936; *p* < 0.0001). The cut-off ADC value with the highest sensitivity and specificity, as determined by the ROC curve, was 1.03 × 10^−3^ mm^2^/s. The ADC was lower than or equal to that cut-off value in 118 lesions, of which 116 (98.3%) were malignant, whereas 7 0 (61.4%) of the 114 in which it was higher than that cut-off value were benign, the difference between the two proportions being statistically significant (*p* < 0.001).

In two cases, the histopathology classified the lesions as benign but the ADC values were indicative of malignancy (false-positive results). In both of those cases, the diagnosis was stromal fibrosis without atypia. In addition, there were 44 lesions classified as malignant in the histopathological analysis but showing ADC values above the cut-off (false-negative results). Among those, the diagnoses were as follows: IC-NST, in 20 patients (45.5%); DCIS, in 9 (20.5%); invasive lobular carcinoma, in 6 (13.6%); carcinoma with intramammary lymph node metastasis, in 1 (2.3%); invasive mucinous carcinoma, in 1 (2.3%); pleomorphic lobular carcinoma, in 1 (2.3%); invasive focal tubular carcinoma, in 1 (2.3%); invasive papillary carcinoma, in 1 (2.3%); mixed ductal and lobular carcinoma, in 1 (2.3%); and metaplastic carcinoma, in 2 (4.5%). When analyzing the histological and immunohistochemical features of the invasive false-negative carcinomas, we found that 91.2% presented histological grade 2 or 3, 69.0% presented nuclear grade 3, and 51.5% were of the luminal B immunophenotype.

Table 3 correlates the BI-RADS classifications, obtained from the evaluation of the morphological and dynamic criteria, with the ADC values, obtained from the DWI, using the cutoff point of 1.03 × 10^−3^ mm^2^/s. Table 4 demonstrates the sensitivity, specificity, PPV, NPV, and accuracy of MRI and DWI, separately and in combination. For the combination of MRI and DWI findings, DWI was considered only in the analysis of the BI-RADS category 4 lesions, as follows:

**Table 3:** Evaluation of diffusion in the lesions, by BI-RADS category and ADC cut-off value (1.03 × 10^-3^ mm^2^/s), in relation to the histological findings.

		Histological result					
		Benign			Malignant		
Category	ADC cut-off	N	(%)		N	(%)	*P *
BI-RADS 2	≤ 1.03 > 1.03	0 2	(0.0) (100)		0 0	(0.0) (0.0)	NP
BI-RADS 3	≤ 1.03 > 1.03	0 37	(0.0) (100)		0 0	(0.0) (0.0)	NP
BI-RADS 4	≤ 1.03 > 1.03	2 31	(22.2) (81.6)		7 7	(77.8) (18.4)	< 0.001
BI-RADS 5	≤ 1.03 > 1.03	0 0	(0.0) (0.0)		23 9	(100) (100)	NP
BI-RADS 6	≤ 1.03 > 1.03	0 0	(0.0) (0.0)		86 28	(100) (100)	NP

NP, not possible (to test the association).

**Table 4:** Sensitivity, specificity, PPV, NPV, and accuracy of MRI using the BI-RADS criteria alone, of qualitative and quantitative DWI evaluations, and of the combination of the two (BI-RADS MRI + quantitative DWI evaluation), for distinguishing between benign and malignant lesions.

	Sensitivity	Specificity	PPV	NPV	Accuracy
BI-RADS MRI	100.0%	54.9%	79.7%	100.0%	83.7%
DWI - qualitative evaluation	97.5%	35.2%	72.7%	88.9%	75.0%
DWI - quantitative evaluation (ADC cut-off: 1.03 × 10^-3^ mm^2^/s)	75.5%	97.2%	98.3%	61,.4%	80.2%
Combination of BI-RADS MRI + DWI - quantitative evaluation	95.7%	96.4%	98.1%	92.0%	95.9%

- BI-RADS 2 or 3, regardless of the DWI findings = probably benign.
- BI-RADS 4, with DWI indicative of benign status = probably benign.
- BI-RADS 4, with DWI indicative of malignant status = suspected malignancy.
- BI-RADS 5 or 6, regardless of the DWI findings = suspected malignancy.

When MRI and DWI were combined, the accuracy of the tests reached 95.9%, with a sensitivity of 95.7% and a specificity of 96.4%.

## DISCUSSION

The capacity of DWI sequences of MRI to characterize the mobility of the water molecules allows indirect evaluation of the microstructure of the tissue by the grading of its cellularity. Based on this principle, it is expected that the use of such sequences will increase the specificity of the method and ultimately decrease the number of unnecessary invasive procedures because of the high sensitivity of the contrast-enhanced images^(13)^. Because DWI sequences are already included in most MRI protocols, they do not entail additional costs; they also have an average acquisition time of less than 5 min.

In the present study, the qualitative evaluation based on DWI alone showed high sensitivity (97.5%), although its low specificity (35.2%) made it incapable of differentiating between malignant and benign lesions in the majority of cases. In the quantitative analysis, malignant lesions showed significantly lower ADC values than did benign lesions. Therefore, the quantitative DWI analysis (ADC measurement) provided a greater contribution to the differentiation between benign and malignant breast lesions in our study.

Chen et al. conducted a meta-analysis to evaluate the performance of the quantitative DWI analysis. They evaluated 964 lesions, of which 615 were malignant and 349 benign, and the mean cut-off ADC values for differentiation ranged from 0.9 × 10^−3^ mm^2^/s to 1.76 × 10^−3^ mm^2^/s, sensitivity and specificity ranging from 63% to 100% and from 46% to 97%, respectively. The mean ADC values ranged from 1.0 × 10^−3^ mm^2^/s to 1.82 × 10^−3^ mm^2^/s for the benign lesions and from 0.87 × 10^−3^ mm^2^/s to 1.36 × 10^−3^ mm^2^/s for the malignant lesions^(9)^. That considerable variation is explained by the different protocols used in the studies. The cut-off ADC values obtained in the differentiation between benign and malignant are dependent upon the respective b values chosen. Therefore, the cut-off value obtained with a b value of 1000 s/mm^2^ cannot be used for lesions evaluated with a b value of 500 s/mm^2^. The results we obtained with a b value of 750 s/mm^2^, in terms of the ADC values, cut-off value, sensitivity, and specificity, are in agreement with those found in the literature.

Despite the promising capacity of ADC values to differentiate between benign and malignant lesions, the ADC values for malignant and benign lesions can overlap, leading to false-positive and false-negative results. Parsian et al.^(14)^ studied benign lesions and found that false-positive results were most often obtained for high-risk lesions, atypical ductal hyperplasia being the most common subtype. Other studies have often reported false-positive results for intraductal papilloma^(15-17)^. In our study, we obtained false-positive results for only two lesions, both of which were subsequently diagnosed as stromal fibrosis without atypia, with ADC values of 0.89 × 10^−3^ mm^2^/s and 0.90 × 10^−3^ mm^2^/s, respectively.

It is known that high levels of ADC are frequently associated with benign changes or benign tumors; although some IC-NSTs show ADC values higher than the cut-off established for malignancy, leading to false-negative results^(15,17)^. The malignant histological subtype with the highest ADC values is mucinous carcinoma, which is characterized by low cellularity and a predominance of mucin, therefore often producing false-negative results in DWI^(18,19)^.

Using the ADC cut-off value established in the present study, we obtained false-negative results in 44 of the 161 malignant lesions evaluated. Among those 44 lesions, the histopathological findings were, in decreasing order of frequency, IC-NST (in 45.5%), DCIS (in 20.5%) and invasive lobular carcinoma (in 13.6%). Of the IC-NSTs for which false-negative results were obtained in DWI, the majority were large tumors with high histological and nuclear grades, as well as high Ki-67 expression, which can be associated with necrosis and edema, factors that are related to an increase in the ADC value. However, the morphological and dynamic characteristics of MRI have high sensitivity and specificity in such cases. Therefore, in the combined evaluation, these tumors would be diagnosed independently of any other additional sequence.

In our study sample, the proportion of false-negative results was higher among the lesions with non-nodular enhancement than among those with nodular enhancement (56.0% vs. 22.2%). Lesions with non-nodular enhancement typically include DCIS, fibrocystic disease, and lobular carcinoma; such lesions can contain areas of normal fibroglandular and adipose tissue (i.e., tissue free of cell hyperproliferation), which can increase the ADC values obtained, thus leading to false-negative results^(20-22)^. High ADC values related to non-nodular enhancement also explain the predominance of false-negative results among the cases of DCIS in our sample. Despite the small number of DCIS cases in our sample (*n* = 11), these tumors showed a mean ADC value higher than that of invasive carcinomas.

The ADC threshold value for the differentiation of benign and malignant breast lesions should be selected according to the purpose of the examination. If the objective is screening with DWI alone, the use of higher ADC threshold values is recommended, as a means of reducing the risk of false-negative results. However, when DWI is used in conjunction with MRI, the use of lower ADC threshold values is recommended, as a means of reducing the risk of false-positive results^(9)^.

Some studies have compared histological grade and tumor biological markers—expression of estrogen and progesterone hormone receptors; expression of human epidermal growth factor receptor 2 (HER2); and the Ki-67 cell proliferation index—with ADC values, attempting to identify associations, although the results have been inconsistent and occasionally contradictory^(23)^. Belli et al.^(24)^ studied 289 patients with malignant carcinoma. Comparing ADC values with the histological subtype and grade, the authors found significant differences between grade 1 carcinoma and grade 2 or 3 carcinoma, as well as between invasive carcinoma and DCIS. Jeh et al.^(25)^ studied 107 cases of IC-NST in correlation with tumor prognostic factors and found ADC values to be significantly lower in tumors that were HER2-negative than in those that were HER2-positive. Mori et al.^(26)^ studied 86 cases of IC-NST and demonstrated a significant difference in ADC values between tumors with high and low Ki-67 indices. Kim et al.^(27)^ studied 67 women with invasive carcinoma and found no significant association between ADC values and tumor prognostic factors, including tumor grade and expression of biological markers.

In the present study, the ADC values in malignant tumors were not found to be significantly associated with histological or immunohistochemical findings related to aggressiveness. However, the invasive malignant lesions in our sample presented similar characteristics regarding histological grade and immunohistochemical profile: 91.9% of the lesions presented histological grade 2 or 3; 74.1% presented nuclear grade 3; and 84.5% were luminal A or luminal B lesions. This relative homogeneity of the histological grade and immunohistochemical profile among the invasive malignant lesions in our sample might have limited the associations with DWI.

When we evaluated the combined use of MRI with DWI compared with MRI alone, we found that their combined use resulted in a significant increase in specificity (96.4%), without a significant reduction in sensitivity (95.7%), corresponding to a significant increase in accuracy (95.9%), confirming our expectations and the data in the literature^(28)^. DWI was particularly useful in cases of lesions categorized as BI-RADS 4, which were responsible for the lower specificity of MRI. Through analysis of the ADC values obtained for the BI-RADS 4 group combined with that of the MRI findings, we were able to propose subdivision of the category BI-RADS 4 into two groups—“probably benign”, comprising lesions with ADC values above the cut-off; and “suspected malignancy”, comprising lesions with ADC values equal to or below the cut-off—with high rates of sensitivity, specificity, and accuracy (95.7%, 96.4%, and 95.9%, respectively).

In the present study, the use of DWI had a greater impact on the evaluation of BI-RADS 4 lesions than on that of lesion in other categories, allowing a better, more complete assessment of these lesions and leading to tailored practices. These findings are in agreement with those of Almeida et al.^(29)^, who demonstrated that DWI can improve the diagnostic performance of MRI and facilitate the division of BI-RADS 4 lesions into the subcategories 4A, 4B, and 4C. In addition, in comparison with MRI alone, MRI plus DWI can more accurately corroborate benign results in BI-RADS 4 lesions, as well as clarifying the analysis of lesions with a discordant histopathological result from a biopsy fragment, leading to a more accurate surgical evaluation.

The results of the present study should be considered in the context of certain limitations. Because it was a retrospective study, many cases could not be evaluated, because it was not possible to recover the MRI data from our digital archive. We were also forced to exclude examinations in which there were technical difficulties in the acquisition of images due to susceptibility artifacts that resulted in image distortion and impaired the characterization of the lesion. It is known that DWI is highly sensitive to such artifacts, and it is hoped that technical innovations currently in development will bring improvements in the resolution of DWI of the breast^(20)^. It is also noteworthy that our patient population, because it comprised individuals treated at a cancer center, featured a predominance of malignant pathological findings, which could have influenced the results.

In conclusion, the findings of the present study demonstrate that the use of DWI can facilitate the characterization of breast lesions, especially those categorized as BI-RADS 4, thus increasing the specificity and diagnostic accuracy of MRI. This method provides greater confidence in the management of this patient population and, after further studies involving larger samples have been conducted, might even be used in order to reduce the number of unnecessary biopsies.
